# Catalpol Exerts a Neuroprotective Effect in the MPTP Mouse Model of Parkinson’s Disease

**DOI:** 10.3389/fnagi.2019.00316

**Published:** 2019-11-15

**Authors:** Li-Yuan Wang, Xin Yu, Xiao-Xi Li, Yi-Nan Zhao, Chun-Yan Wang, Zhan-You Wang, Zhi-Yi He

**Affiliations:** ^1^Department of Neurology, the First Hospital of China Medical University, Shenyang, China; ^2^Institute of Health Science, China Medical University, Shenyang, China

**Keywords:** Parkinson’s disease, catalpol, neuroprotection, apoptosis, inflammation

## Abstract

The degeneration of dopaminergic (DA) neurons in Parkinson’s disease (PD) is related to inflammation and oxidative stress. Anti-inflammatory agents could reduce the risk or slow the progression of PD. Catalpol, an iridoid glycoside extracted from the roots of *Rehmannia* radix, has been reported to reduce the release of inflammatory factors and exert neuroprotective effects. 1-methyl-4-phenyl-1,2,3, 6-tetrahydropyridine (MPTP)-treated mice were used as the PD model and the roles of catalpol on DA neurons and its potential mechanism were investigated in this study. We found that catalpol administration mitigated the loss of DA neurons induced by MPTP and increased exploratory behavior along with tyrosine hydroxylase (TH) expression, which was accompanied by astrocyte and microglia activation. Importantly, catalpol administration significantly inhibited MPTP-triggered oxidative stress, restored growth-associated protein 43 (GAP43) and vascular endothelial growth factor (VEGF) levels. Further, we found that catalpol suppressed the activation of MKK4/JNK/c-Jun signaling, and reduced the pro-inflammatory factors and inflammasome in the mouse model of PD. Our results suggest that catalpol relieves MPTP-triggered oxidative stress, which may benefit to avoid the occurrence of chronic inflammatory reaction. Catalpol alleviates MPTP-triggered oxidative stress and thereby prevents neurodegenerative diseases-related inflammatory reaction, highlighting its therapeutic potential for the management of PD symptoms.

## Introduction

Parkinson’s disease (PD) is a common and complex age-related neurological disorder of unclear etiology. The clinical symptoms of PD include motor dysfunction, resting tremor, postural and gait disorders, and neurological impairment (Kalia and Lang, 2015). The pathological hallmarks of PD are the loss of dopamine (DA) neurons and the presence of Lewy bodies, with a resultant reduction in dopamine levels in the substantia nigra (SN) pars compacta and striatum (Bendor et al., 2013; Kordower et al., 2013). Although the underlying molecular mechanisms are not well understood, apoptosis, neuroinflammation, and oxidative stress are thought to be involved in these effects (Imamura et al., 2003; Ghavami et al., 2014; Mashima et al., 2018). For example, activated microglia was shown to contribute to the degeneration of DA neurons in animal models of PD (Gao et al., 2002). Neuroinflammation plays an important role in PD progression (Wahner et al., 2007). Oxidizing agents could induce the death of DA neurons (Carr et al., 2000; Szabó et al., 2007). Some therapeutic schemes for increasing dopamine levels have not achieved satisfactory results in alleviating clinical symptoms defects, which is considered to be related to the poor effects on reducing dopaminergic neuron damage (Liu et al., 2018; da Silva et al., 2018). Hence, there is an urgent need to seek new drugs with multiple potentials in alleviating the death of DA neurons in PD.

Catalpol, a water-soluble active compound isolated from *Rehmannia glutinosa*, has many pharmacological activities including antiapoptotic, anti-inflammatory and antioxidant functions as well as neuroprotective effects (Hu et al., 2010; Wang and Zhan-Sheng, 2018; Yan et al., 2018). Catalpol can cross the blood-brain barrier and serve as a protective agent in the treatment of neurodegenerative diseases (Wang et al., 2012; Dinda et al., 2019). Catalpol exposure reduced tumor necrosis factor-α (TNF)-α and nitric oxide synthase expression in cultured primary microglia that was upregulated by the inflammatory primer, lipopolysaccharide (LPS; Tian et al., 2006), and also protected astrocytes from oxidative damage caused by hydrogen peroxide (Bi et al., 2008a). In other studies, catalpol prevented 1-methyl-4-phenyl-1,2, 3,4-tetrahydropyridine (MPTP)/1-methyl-4-phenylpyridinium-induced neurotoxicity in mesencephalic neurons not only by enhancing antioxidant activity, but also by inhibiting monoamine oxidase type B-mediated–neurotoxicity in cells (Mao et al., 2007; Tian et al., 2007; Bi et al., 2008b, 2009; Chen et al., 2008), it also restored locomotor function by increasing the striatal DA concentration and enhanced the level of glial cell-derived neurotrophic factor, thereby preventing the loss of tyrosine hydroxylase (TH)-positive neurons and downregulation of striatal DA transporter (DAT) in an MPTP-induced PD model (Xu et al., 2010). Moreover, catalpol protected against rotenone-induced cell damage by increasing the activities of complex I, glutathione (GSH), GSH peroxidase (GPx) and superoxide dismutase (SOD), which reduced lipid peroxidation and preserved mitochondrial membrane potential (Mao et al., 2007). However, the molecular basis of these effects remains unclear.

In this study, we investigated the neuroprotective effects of catalpol on DA neurons and analyzed its underlying mechanisms using a mouse model of PD. Catalpol treatment prevented DA neurons against apoptosis by downregulating the mitogen-activated protein kinase kinase (MKK) 4/c-Jun N-terminal kinase (JNK)/c-Jun signaling pathway in MPTP-treated mice. Furthermore, catalpol not only reduced the levels of pro-inflammatory factors and inflammasome activation, but it also inhibited oxidative stress in the SN.

## Materials and Methods

### Animals and Drug Treatments

**Figure 1:**
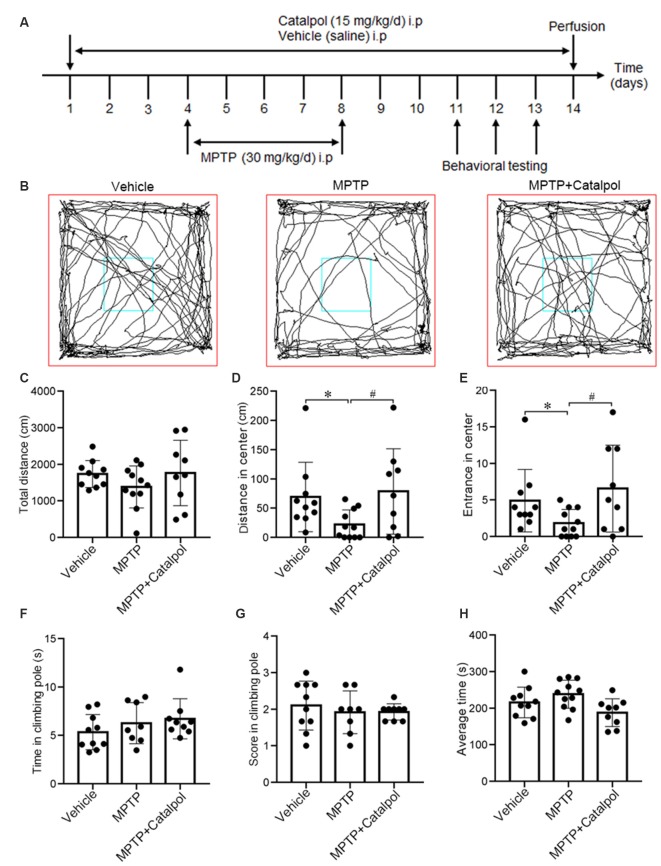
Catalpol alleviates impairment of exploratory behavior in 1-methyl-4-phenyl-1,2,3,6-tetrahydropyridine (MPTP)-treated mice. Mice were intraperitoneally injected with catalpol (15 mg/kg/day) or vehicle (saline) for 3 days, and then administered MPTP (30 mg/kg/day; *n* = 11), MPTP + catalpol, or vehicle starting on day 4 for 5 days. Mice that were previously treated with MPTP + catalpol (*n* = 9) were continually administered catalpol for 6 days; those primed with MPTP received the vehicle for 6 days. Mice treated with the vehicle saline served as the control group (*n* = 10). **(A)** Treatment schedule. **(B)** Representative images of movement trials in the open field test (OFT). **(C)** Quantitative analysis of total distance traveled in the OFT. **(D)** Distance from the zone center. **(E)** The number of entries into the zone center in the OFT. **(F,G)** Time and score in the pole-climbing test. **(H)** The average amount of time mice remained on the rod in the Rotarod test. Data represent mean ± SEM. The *P*-values were calculated using a one-way analysis of variance (ANOVA). *P < 0.05 vs. vehicle group; ^#^*P* < 0.05 vs. MPTP-induced group.

Male C57BL/6 mice (10 weeks old) were obtained from the Department of Laboratory Animal Science of China Medical University. The mice (*n* = 11 pre-group) were intraperitoneally injected with catalpol dissolved in saline (15 mg/kg/day; Chengdu Manster Biotechnology Co., Chengdu, China; A0215) or vehicle (saline) for 3 days, followed by MPTP (30 mg/kg/day; Sigma-Aldrich, St. Louis, MO, USA; M0896), MPTP + catalpol, or vehicle starting on day 4 for 5 days. Mice in the MPTP + catalpol treatment group (*n* = 9) were continually administered catalpol for 6 days, and those primed with MPTP received the vehicle for 6 days. Mice in the control group were given the vehicle saline (*n* = 10). The experimental procedure is outlined in Figure 1A. The general health and body weight of animals were monitored daily, and animals were handled according to the Guide for the Care and Use of Medical Laboratory Animals (Ministry of Health, Beijing, China). The experimental protocol was approved by the Laboratory Ethics Committee of China Medical University.

### Open Field Test (OFT)

The open field test (OFT) is used to assess spontaneous motor function and exploratory behavior. Mice were allowed to adapt to the new environment before beginning the test, as previously described (Zhang Y. H. et al., 2018). A mouse was placed in the center of the arena and the whole experiment lasted 5 min. The exploratory trace, total distance, distance in the zone center and the times that the mouse enters into the center of the arena were recorded using SMART v.3.0 software (Harvard Apparatus, British).

### Pole-Climbing Test

A 50-cm pole was split in half, with each section measuring 25 cm. The middle of the pole was marked. A small ball was placed at the top of the pole and covered with gauze to prevent it from slipping. The mouse was placed on the bar and the time taken until it reached the marked line was recorded. The scores were determined as follows: >6 s, 1 point; 3–6 s, 2 points; and <3 s, 3 points.

### Rotarod Test

Locomotor ability was tested using a five-lane IITC 755 Series 8 rotarod (IITC Life Science, Woodland Hills, CA, USA) that was rotated starting at a speed of 4 rpm, which was increased to 40 rpm over a period of 5 min in forwarding mode with a 5-min rest interval. Each mouse was tested three times at each time point.

### Tissue Preparation

After behavioral testing, mice were anesthetized by intraperitoneal injection of sodium pentobarbital at a dose of 40 mg/kg. They were then perfused with saline and sacrificed by decapitation. Half of the brain was fixed with 4% paraformaldehyde for morphological analysis. The SN and striatum of the other half of the brain were flash-frozen in dry ice and stored at −80°C until biochemical analyses.

### Reactive Oxygen Species (ROS) Measurement

To determine whether catalpol treatment decreases oxidative stress, we detected ROS levels in the SN with the Reactive Oxygen Species Assay Kit (Beyotime Institute of Biotechnology, Shanghai, China; S0033). Briefly, the SN was weighed and homogenized in ice-cold phosphate-buffered saline, then centrifuged at 500× *g* for 10 min at 4°C. The supernatant was used for the ROS assay. ROS production was evaluated using the oxidant-sensitive probe 2,7-dichlorofluorescein diacetate according to the manufacturer’s instructions. Fluorescence intensity was measured at 485 nm excitation and 525 nm emission wavelengths using a microplate reader (Cytation5; BioTek, Winooski, VT, USA). ROS level is expressed as fluorescence intensity per microgram of protein.

### Determination of SOD Activity

The SOD activity was measured by ultraviolet spectroscopy with the SOD Assay Kit (Jiancheng Biology, Nanjing, China; A001–1) according to the manufacturer’s instructions. Briefly, the SN was removed from the mouse brain and weighed and homogenized in ice-cold SOD sample preparation solution. The lysate was centrifuged at 4,000× *g* for 10 min at 4°C, and the supernatant was used for the assay.

### Western Blot Analysis

Mouse brain tissue was collected and homogenized by sonication in ice-cold RIPA buffer (Beyotime Institute of Biotechnology; P0013B) containing a protease inhibitor cocktail (Sigma-Aldrich, St. Louis, MO, USA; 8340) for 3 h at 4°C. After centrifugation at 12,000× *g* at 4°C for 20 min, protein concentration in the supernatant was determined with bicinchoninic acid protein assay reagent (Beyotime Institute of Biotechnology; P0010). The supernatant containing 25 μg of protein was resolved by 12% sodium dodecyl sulfate-polyacrylamide gel electrophoresis, and the proteins were transferred to a polyvinylidene difluoride membrane (Millipore, Billerica, MA, USA; IPVH00010) that was probed with the following antibodies: rabbit anti-TH (1:1,000, Millipore); rabbit anti-DAT (1:500), rabbit anti-SOD1 (1:500), rabbit anti-GPx4 (1:500), mouse anti-glyceraldehyde 3-phosphate dehydrogenase (GAPDH; 1:10,000), rabbit anti-β-actin (1:10,000), rabbit anti-growth-associated protein (GAP) 43 (1:500), and rabbit anti-vascular endothelial growth factor (VEGF; 1:500; all from Proteintech, Rosemont, IL, USA); mouse anti-B cell lymphoma (Bcl) 2 (1:1,000), mouse anti-Bcl2-associated × protein (BAX; 1:1,000), rabbit anti-caspase-3 (1:1,000), mouse anti-caspase-9 (1:1,000), rabbit anti-p-MKK4 (1:1,000), rabbit anti-MKK4 (1:1,000), rabbit anti-p-c-Jun (1:1,000), rabbit anti-c-Jun (1:1,000), rabbit anti-p-JNK (1:1,000), rabbit anti-JNK (1:1,000), rabbit anti-nucleotide-binding domain (NBD) and leucine-rich-repeat-containing (LRR; known as NLR family member) × 1 (NLRX1; 1:1,000), rabbit anti-IL-1β (1:1,000), and rabbit anti-glial fibrillary acidic protein (GFAP; 1:1,000; all from Cell Signaling Technology, Danvers, MA, USA); mouse anti-ionized calcium binding adaptor molecule (Iba)1 (1:1,000, Thermo Fisher Scientific, Waltham, MA, USA); and rabbit anti-α-synuclein (1:1,000), rabbit anti-TNF-α (1:500), and rabbit anti-nucleotide binding oligomerization domain-like receptor, pyrin domain-containing (NLRP) 3 (1:1,000; all from Abcam, Cambridge, MA, USA). The membrane was incubated with horseradish peroxidase-labeled secondary antibodies (1:10,000, Thermo Fisher Scientific, Waltham, MA, USA), and protein band intensity was quantified using Prism v.7.0 software (Graph Pad, La Jolla, CA, USA).

### Immunohistochemistry and Immunofluorescence

Brain tissue sections were incubated overnight at 4°C with rabbit anti-TH antibody (1:100), then treated with the appropriate biotinylated secondary antibody for 2 h at room temperature followed by a third antibody for 30 min (MXB, Fuzhou, China). The sections were developed in diaminobenzidine (Sigma-Aldrich, St. Louis, MO, USA) for 3–5 min, then immersed in distilled water to halt the reaction. The sections were dehydrated and sealed with neutral gum and images were acquired on a light microscope (ECHO, San Diego, CA, USA). For immunofluorescence labeling, mouse brain sections were blocked with sheep serum (Beyotime Institute of Biotechnology) at room temperature for 1 h, then incubated overnight at 4°C with a mixture of primary antibodies consisting of mouse anti-GFAP (1:100) and rabbit anti-TH (1:100), or mouse anti-TH (1:100; ImmunoStar, Hudson, WI, USA) and rabbit anti-Iba1 (1:400; Wako Pure Chemical Industries). After rinsing, the sections were incubated with goat anti-rabbit IgG (H + L) and goat anti-mouse IgG (H + L) cross-adsorbed secondary antibodies (both 1:500, Thermo Fisher Scientific, Waltham, MA, USA) for 2 h at room temperature. Images were acquired with a confocal laser scanning microscope (A1; Nikon, Tokyo, Japan).

### Statistical Analysis

Data are represented as mean ± standard error of the mean (SEM), and each set of experiments was repeated three times in parallel. Differences among means were compared using one-way ANOVA. Data were analyzed using Prism v.7.0 software. The results were considered significant at *P* < 0.05.

## Results

### Catalpol Enhances Exploratory Behavior in MPTP-Treated Mice

To investigate whether catalpol alters the motor function and exploratory behavior in MPTP-treated mice, we used the OFT, pole climbing test and Rotarod test to assess motor ability. The distance traveled and the number of entries in the zone center were greater in the MPTP + catalpol group than in the MPTP group, whereas total distance traveled was similar across groups (Figures 1B–E). We then assessed the effect of catalpol on locomotion ability with the pole climbing and Rotarod tests but found no significant differences in scores between the three groups in either test (Figures 1F–H). These results indicate that catalpol reverses the reduction in exploratory behavior caused by MPTP treatment. MPTP or catalpol treatment did not markedly affect the bodyweight of the mice (Supplementary Figure S1).

### Catalpol Protects DA Neurons From MPTP-Induced Apoptosis

**Figure 2:**
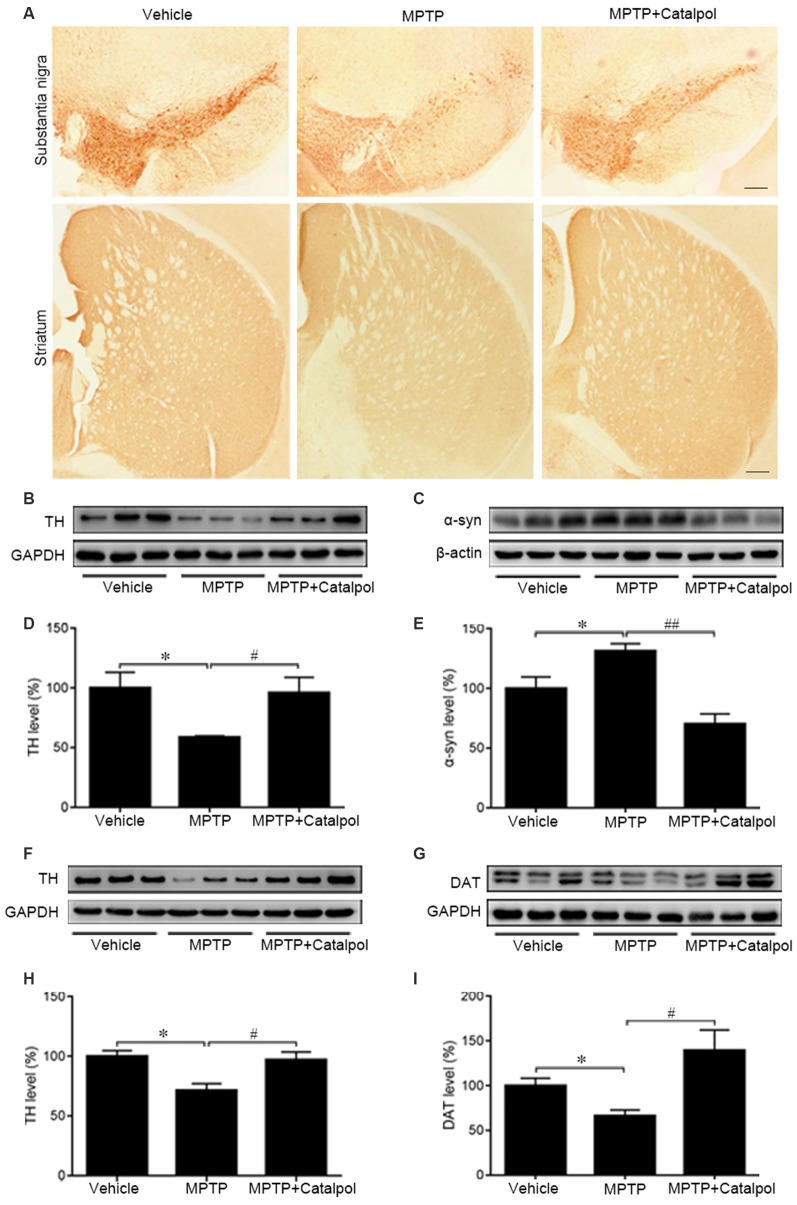
Catalpol rescues dopaminergic (DA) neurons in the SN and striatum in an MPTP-induced mouse model of Parkinson’s disease (PD). **(A)** Immunohistochemical detection of tyrosine hydroxylase (TH) in DA neurons of the substantia nigra (SN) and striatum of mice treated with vehicle, MPTP, or MPTP + catalpol. Scale bar = 100 μm. **(B–E)** Western blot analysis of TH and α-synuclein (α-syn) levels in the SN. **(F–I)** Protein levels of TH and DA transporter (DAT) as detected by western blotting. Data represent mean ± SEM; *n* = 7 mice per group. The *P*-values were calculated using one-way ANOVA. *P < 0.05 vs. vehicle group; ^#^*P* < 0.05, ^##^*P* < 0.01 vs. MPTP-induced group.

Given the massive loss of DA neurons in the SN and striatum of MPTP-treated mice (Kordower et al., 2013), we examined whether catalpol protects DA neurons in the SN and striatum against damage caused by MPTP. TH immunoreactivity was reduced in the SN and striatum of MPTP-treated mice relative to the vehicle group, indicating a loss of DA neurons and nerve fibers; however, catalpol administration reversed this effect (Figure 2A). To confirm this observation, we examined TH and DAT protein levels in the SN and striatum by western blot. Consistent with the results obtained by immunohistochemistry, we found that catalpol treatment increased TH and DAT protein expression compared to MPTP-treated mice (Figures 2B–G) and abrogated the accumulation of α-synuclein in the SN (Figures 2H,I). These results demonstrate that catalpol mitigates the loss of DA neurons caused by MPTP.

**Figure 3:**
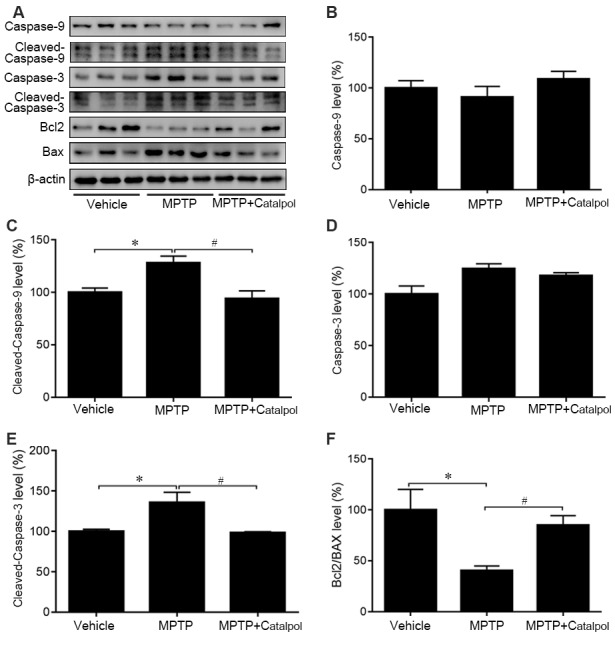
Catalpol inhibits apoptosis in the SN of mice treated with MPTP. **(A)** Western blot analysis of caspase-9, cleaved caspase-9, caspase-3, cleaved caspase-3, Bcl2, and BAX levels in the SN. **(B–F)** Quantitative analysis of caspase-9, cleaved caspase-9, caspase-3, and cleaved caspase-3 levels and Bcl2/BAX ratio. Data represent mean ± SEM; *n* = 8 mice per group. The *P*-values were calculated using one-way ANOVA. *P < 0.05 vs. vehicle group; ^#^*P* < 0.05 vs. MPTP-induced group.

MPTP-induced death of DA neurons has been suggested to result from increased release of the proapoptotic factors caspase-3, caspase-9, and Bax and a decrease in the antiapoptotic factor Bcl2 (Lee et al., 2011). To determine whether catalpol affects apoptosis, we examined caspase-3, caspase-9, Bax and Bcl2 protein expression by western blotting. Cleaved caspase-3 and cleaved caspase-9 levels were elevated in the brain of MPTP-treated mice relative to controls, but this was abolished by catalpol treatment (Figures 3A–E). Accordingly, the decrease in the Bcl2/Bax observed upon MPTP treatment was reversed by catalpol administration (Figures 3A,F). Thus, catalpol prevents the loss of DA neurons by suppressing apoptosis.

### Catalpol Modulates MKK4/JNK/c-Jun Signaling to Inhibit the Apoptosis of DA Neurons

**Figure 4:**
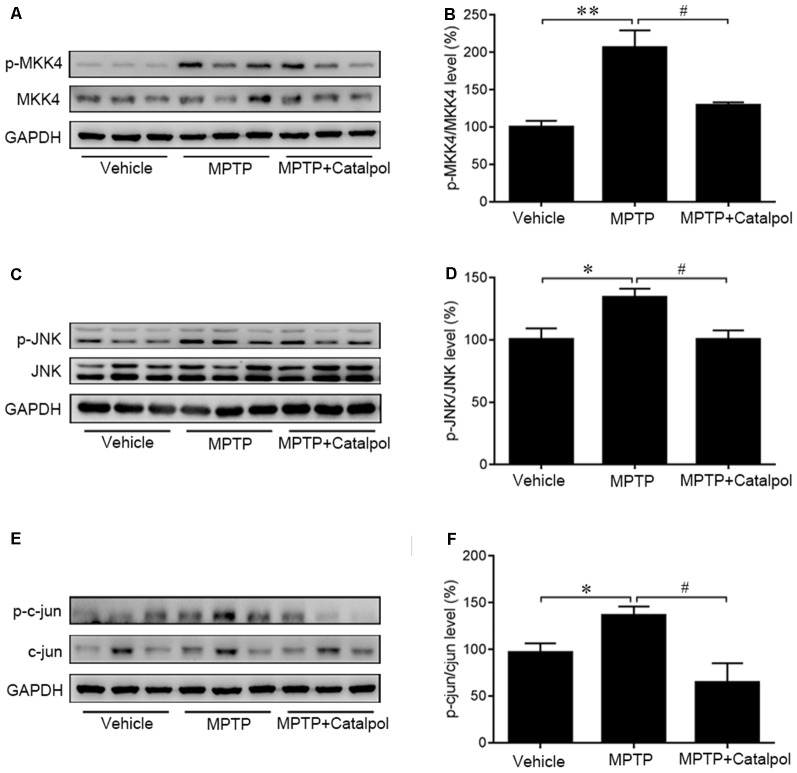
Effects of catalpol on MKK4/JNK/c-Jun signaling in the brain of mice treated with MPTP. **(A,C,E)** Expression of MKK4, phosphorylated (p-) MKK4, JNK, p-JNK, c-Jun, and p-c-Jun detected by western blotting. **(B,D,F)** Quantitative analysis of MPTP-induced changes in protein expression and their reversal by catalpol treatment. Data represent mean ± SEM; *n* = 6 mice per group. The *P*-values were calculated using one-way ANOVA. *P < 0.05, **P < 0.01 vs. vehicle group; ^#^*P* < 0.05 vs. MPTP-induced group.

As JNK/c-Jun signaling is important for apoptosis, we next examined whether catalpol inhibits apoptosis by regulating this pathway. A western blot analysis revealed that MPTP increased JNK and c-Jun phosphorylation, which was reversed by catalpol (Figures 4C–F). Given that MKK4 signaling is a key regulator of JNK activation, we evaluated MKK4 phosphorylation status in the SN and determined that it was altered by catalpol, although total MKK4 protein level was unchanged compared to the MPTP-treated group (Figures 4A,B). These findings indicate that catalpol blocks apoptosis *via* modulation of MKK4/JNK/c-Jun signaling.

### Catalpol Exerts Antioxidant Effects in MPTP-Treated Mice

**Figure 5:**
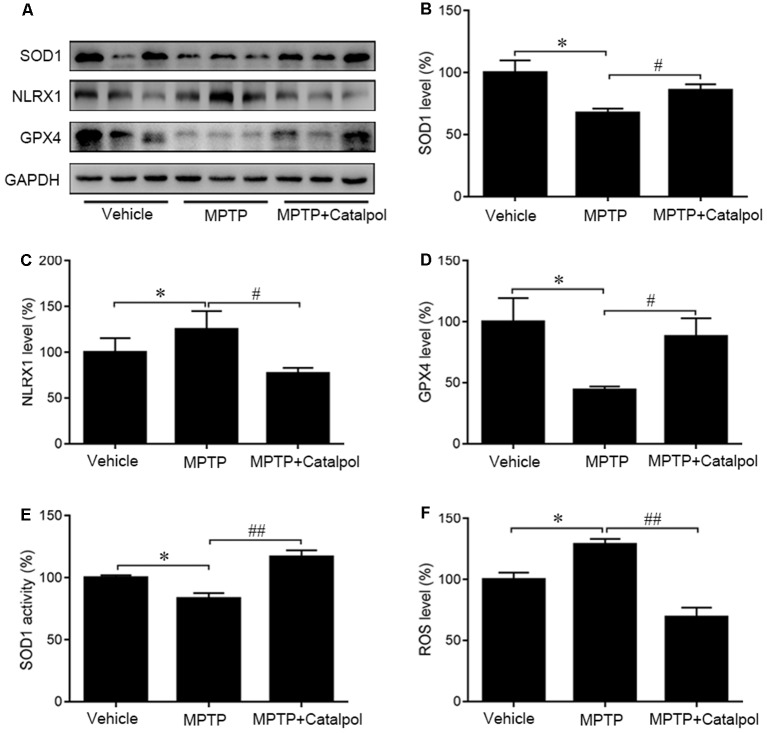
Protective effects of catalpol against oxidative stress in the SN of MPTP-treated mice. **(A)** SOD1, NLRX1, and GPX4 protein expression as determined by western blotting. Glyceraldehyde 3-phosphate dehydrogenase (GAPDH) served as the loading control. **(B–D)** Quantitative analysis of SOD1, NLRX1, and GPX4 protein levels. **(E)** Changes in SOD1 activity in the SN of mice. **(F)** Reactive oxygen species (ROS) production in the SN detected with the dichlorofluorescein diacetate probe. Data represent mean ± SEM; *n* = 6–8 mice per group. The *P*-values were calculated using one-way ANOVA. *P < 0.05 vs. vehicle group; ^#^*P* < 0.05, ^##^*P* < 0.01 vs. MPTP-induced group.

Oxidative stress is a major cause of apoptosis in DA neurons (Haddad, 2002), we, therefore, evaluated ROS levels in the SN and found that MPTP enhanced ROS levels (Figure 5A). ROS levels were decreased in the presence of catalpol, which also reversed the MPTP-induced suppression of SOD1 activity and protein expression in the SN (Figures 5B–D). Moreover, western blot analysis showed that catalpol treatment restored the expression of the antioxidant enzyme GPx4, which was downregulated in the presence of MPTP (Figures 5C,E). Given that NLRX1 enhances ROS production, we examined whether catalpol-mediated inhibition of ROS is involved in the suppression of NLRX1 and found that expression of the protein was reduced by treatment with catalpol relative to the level in mice treated with MPTP only (Figures 5B,F). These results indicate that catalpol alleviates the apoptosis of DA neurons through its antioxidant capacity.

### Catalpol Attenuates Inflammation in MPTP-Injured Mice

**Figure 6:**
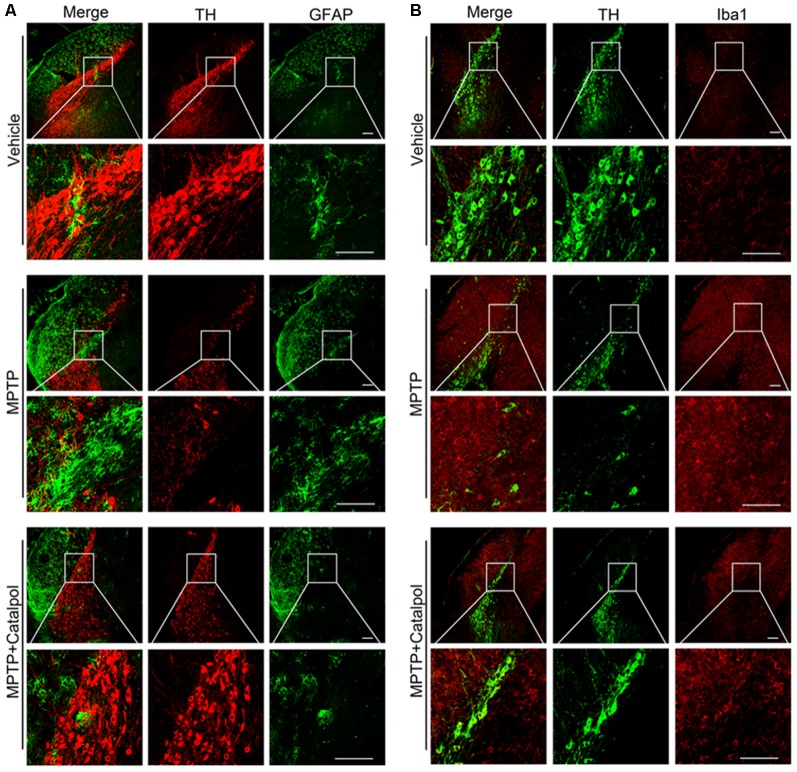
Catalpol treatment suppresses microglia and astrocyte activity induced by MPTP. **(A)** Frozen sections of the SN were labeled with anti-TH (red) and anti-GFAP (green) antibodies to detect DA neurons and astrocyte activity, respectively. Scale bar = 100 μm. **(B)** Brain sections were labeled with anti-TH (green) and anti-Iba1 (red) antibodies to assess microglia activity in the SN. Scale bar = 100 μm; *n* = 6.

**Figure 7:**
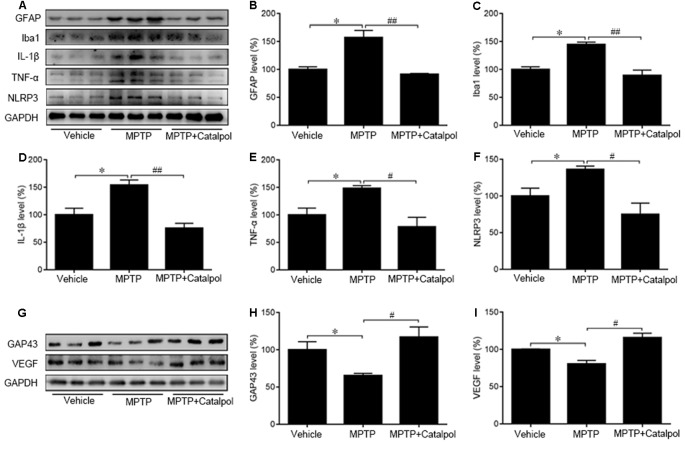
Catalpol suppresses MPTP-induced inflammation and promotes neural regeneration in the SN of mice. **(A)** Western blot analysis of GFAP, Iba1, IL-1β, TNF-α, and NLRP3 protein expression in the SN of mice. **(B–F)** Catalpol blocks MPTP-induced astrocyte and microglia activation inhibits IL-1β and TNF-α, and suppresses NLRP3 production. **(G–I)** Growth-associated protein 43 (GAP43) and vascular endothelial growth factor (VEGF) protein expression detected by western blotting. Data represent mean ± SEM; *n* = 7 mice per group. The *P*-values were calculated using one-way ANOVA. *P < 0.05 vs. vehicle group; ^#^*P* < 0.05, ^##^*P* < 0.01 vs. MPTP-induced group.

PD development and progression are accompanied by an inflammatory response. To determine whether catalpol reduces inflammation in PD, we evaluated the activation astrocyte and microglia based on immunohistochemical detection of GFAP and Iba1 expression, respectively. The immunopositivity of GFAP and Iba1 was increased in mice treated with MPTP compared to the vehicle. However, catalpol had the opposite effect (Figures 6A,B). Consistent with these findings, the western blot analysis showed that GFAP and Iba1 protein levels were elevated in MPTP-treated mice, but these were blocked by catalpol (Figures 7A–C). We also found that catalpol inhibited MPTP-induced inflammation as evidenced by the downregulation of TNF-α, interleukin (IL)-1β and NLRP3 protein expression compared to mice treated with MPTP only (Figures 7A,D–F).

GAP43 promotes nerve regeneration and the establishment of synaptic connections (Allegra Mascaro et al., 2013; Kettenmann et al., 2013). Moreover, the inhibition of GAP43 may be due to the increase of apoptosis (Wang Z. et al., 2018). To assess MPTP-induced injury of DA neurons and to investigate whether catalpol is beneficial for regeneration, we determine by western blotting that GAP43 protein expression was lower in mice treated with MPTP as compared to vehicle (Figures 7G,H). However, catalpol administration restored GAP43 protein level. In addition to its proangiogenic role, VEGF also promotes neuronal survival (Greenberg and Jin, 2005; Okabe et al., 2014); here we found that MPTP reduced VEGF protein expression, which was restored by catalpol (Figures 7G,I). These data indicate that catalpol protects DA neurons against MPTP-induced injury by inhibiting inflammation and stimulating neural regeneration.

## Discussion

Inflammation is implicated in the pathogenesis of PD (Liu et al., 2003; Herrero et al., 2015; Ransohoff, 2016) increases in innate immune components such as interleukin (IL)-1β, IL-6 and TNF-α have been detected in the cerebrospinal fluid and SN of PD patients (Fiszer et al., 1994; Liu et al., 2003). Additionally, in postmortem studies and animal models of PD, ROS and nitrogen species were found to be increased, which was accompanied by activation of microglia and reactive astrocytes (Herrero et al., 2015; Ransohoff, 2016). Serum proinflammatory cytokine concentrations are also increased in PD patients (Dobbs et al., 1999; Scalzo et al., 2010). In rodents, inflammatory stimuli including bacterial LPS (Qin et al., 2007) and viral pathogens (Ogata et al., 1997) contribute to the loss of DA neurons. Bacterial or viral infection may increase the onset of PD in a susceptible population (Jang et al., 2009). In animal models of MPTP-induced PD (Dauer and Przedborski, 2003), TNF-α level in the brain was increased, implying that adaptive immunity was activated in response to MPTP-induced neurodegeneration (Barcia et al., 2011). Peripheral and/or central neuroinflammation is associated with the prodromal phase of PD (Hirsch and Hunot, 2009); thus, inflammation is an important consideration when investigating the mechanisms underlying the loss of DA neurons in PD. Interestingly, LPS can cause prolonged activation of MKK4 (Waetzig et al., 2005), leading to the phosphorylation of JNK, which mediates the proinflammatory function of microglia (Verrecchia et al., 2003; Waetzig et al., 2005). Phosphorylated JNK stimulates c-Jun activity to enhance the transcription of pro-apoptotic cytokines (Whitfield et al., 2001; Dunn et al., 2002). It was reported that anti-inflammatory intervention can reduce the risk of developing PD (Noyce et al., 2012), while pharmacological or genetic inhibition of inflammatory factors conferred protection against MPTP-induced neurodegeneration (Du et al., 2001; Nomura et al., 2011; Świątkiewicz et al., 2013). The bioflavonoid compound pycnogenol, which is commonly used as a dietary supplement to suppress inflammation, alleviated MPTP-associated motor impairment and the inflammatory responses of astrocytes and microglia in a mouse model of PD (Khan et al., 2013). Likewise, minocycline which has anti-inflammatory properties blocked the activation of macrophages in the brain to protect against apoptosis *in vitro* (Duan et al., 2002) and in animal models of PD (Du et al., 2001; Wu et al., 2002). Thus, neuroinflammation is a major mechanism underlying PD, and early intervention with anti-inflammatory agents is a potential strategy for inhibiting PD progression. It is worth noting that Dinda et al. (2019) compared the effects of catalpol, geniposide, and harpagoside. Catalpol exhibits significant advantages of neuroprotective potential in the intervention of PD (Dinda et al., 2019). Catalpol could protect the neurons from MPP^+^-induced toxicity *in vitro* (Tian et al., 2006). Catalpol can pass the blood-brain barrier. Catalpol treatment markedly protected the neurons of PD mice brain from degeneration through improving antioxidant status (Mao et al., 2007). Catalpol obviously mitigated the loss of dopamine transporter density and TH positive neurons and improved the locomotor ability of PD mice (Xu et al., 2010). We previously reported that catalpol could alleviate β-amyloid toxicity and neuroinflammatory reactions (Wang N. et al., 2018). It is considered that catalpol has the potential property of slowing down the neurodegenerative process in PD.

In this study, the inflammatory response in the mouse brain was accompanied by MPTP-induced neuronal apoptosis, as evidenced by the upregulation of cleaved caspase-3 and cleaved caspase-9 both effects in the extrinsic apoptosis pathway (Zou et al., 1997) relative to vehicle-treated control animals. Additionally, the decrease in Bcl2/Bax ratio upon MPTP treatment indicated that the intrinsic apoptosis pathway was activated (Falschlehner et al., 2007). The presence of reactive astrocytes and activated microglia confirmed MPTP-induced inflammation in our mouse model of PD, whereas the downregulation of GAP43 and VEGF proteins reflected MPTP-mediated neurodegeneration. It is generally accepted that GAP43 is an important biomarker for adult neurogenesis. GAP43 decline is related to the increase of apoptotic cells (Wang Z. et al., 2018). Meanwhile, the behavioral deficits observed in MPTP-treated mice were consistent with nigrostriatal DA neuron injury. The schemes of MPTP-caused PD-like pathology include three types: acute, subacute, and chronic (Petroske et al., 2001; Selvakumar et al., 2014; Ren et al., 2015). The subacute model is often chosen to investigate the effects of interventions due to its advantages in the period and the similarities to PD. Whereas, MPTP subacute administration-induced alterations and the proper positive control are still needed to be further confirmed. In the present study, the subacute MPTP paradigm failed to trigger the typical movement deficits despite the dopaminergic neuron damage. Instead of motor defects, the results of the OFT in our study showed that MPTP-managed mice exhibited behavioral defects relative to controls. Nonmotor characteristics of PD with nigrostriatal circuit degeneration perform anxiety-like behaviors (Santiago et al., 2010). So, in the present study, it is important to assess the validity of a candidate based on its effects on the dopaminergic neurons and behavioral examination related to nonmotor characteristics of PD. It is reported that catalpol reduced inflammatory cytokine levels and improved cholinergic function in a mouse model of senescence (Zhang et al., 2013), and protected pre-myelinating oligodendrocytes against ischemia-induced injury *in vitro* (Cai et al., 2016) while alleviating atherosclerosis (Zhang Y. et al., 2018) and modulating Bcl-2/Bax ratio to suppress endothelial cells apoptosis induced by hydrogen peroxide (Hu et al., 2010). We selected catalpol as our candidate for PD intervention. It has been reported that MPTP treatment could cause olfaction dysfunction, gastrointestinal injury, bladder impairment, and other nonmotor symptoms except for near-total damage of the striatal dopaminergic input (Taylor et al., 2010; Duty and Jenner, 2011). Several studies have shown that antioxidants pretreatment prior to the induction of PD with MPTP could provide more effective neuroprotection against the cytotoxic effects (Lee et al., 2011; Wang et al., 2015). We then administrated the mice by intraperitoneal injection with catalpol ahead of MPTP treatment. Consistent with previous studies (Bi et al., 2008b; Jiang et al., 2008), we demonstrated that catalpol has neuroprotective effects in PD, as evidenced by the restoration of DA neurons in the SN and striatum of MPTP-treated mice and the corresponding increase in exploratory behavior. Immunohistochemical detection of TH showed that catalpol intervention prevented MPTP-induced loss of TH+ cells, suggesting that it can improve the TH deficiency observed in PD patients (Moore et al., 2005). Catalpol treatment alleviated the MPTP-induced downregulation of GAP43 and VEGF proteins, which suggested that catalpol may induce regeneration. Although no significant differences were observed in the scores during the pole climbing and rotarod tests among the vehicle-, MPTP- and MPTP plus catalpol-treated mice, in the OFT, MPTP-lesioned mice exhibited significant thigmotaxis, traveling a shorter total distance and passing fewer times through the center of the open field relative to the vehicle group, whereas, these behavioral impairments were abolished by catalpol treatment. Our findings demonstrated that catalpol could improve some behavioral defects in PD. Xu et al. (2010) observed significant locomotor impairments in the MPTP-triggered PD mouse model, and catalpol treatment could improve the motor performances of these mice. Those incongruences are in contrast with our results perhaps because of the differences in the PD mouse model type (an MPTP-induced chronic paradigm), the dose of catalpol intervention (high dose at 50 mg/kg) and an extended period of time (8 weeks of catalpol treatment) in Xu’s study.

In obese mice, catalpol was shown to mitigate inflammation by blocking JNK signaling (Santiago et al., 2010). In the present study, MPTP induced an increase in MKK4 and JNK phosphorylation, which was accompanied by dopamine depletion in a mouse model of PD. The increased phosphorylation of c-Jun in the brain of MPTP-treated mice suggested an enhancement of c-Jun transcription through JNK activation (Zhou et al., 2015) as previously reported (Derijard et al., 1994), suggesting that MPTP-induced MKK4/JNK/c-Jun signaling is involved in DA neurons degeneration in PD. Catalpol treatment reversed the MPTP-induced phosphorylation of MKK4 and JNK and c-Jun expression, suggesting that it suppresses MKK4/JNK/c-Jun signaling. Given the critical role of JNK in linking inflammation and apoptosis (Dunn et al., 2002; Waetzig et al., 2005; Perier et al., 2007), catalpol may prevent neurodegeneration by targeting this pathway.

Oxidative stress promotes inflammation and causes cell damage (Haddad, 2002). In general, inflammation is a protective response against cell injury that involves the destruction and removal of detrimental components to restore cellular function. DA neurons are highly susceptible to ROS-mediated injury (Miyazaki and Asanuma, 2008); on the other hand, ROS activate pro-inflammatory signaling in glia, thereby stimulating a sustained inflammatory response. Chronic inflammation ultimately destroys normal tissue. TNF-α, which is released by reactive astrocytes and activated microglia, modulates oxidative stress and inflammation in neurodegenerative diseases (Fischer and Maier, 2015). Elevated TNF-α levels due to systemic inflammation are associated with the decline of DA neurons in PD (Sriram et al., 2006; Allegra Mascaro et al., 2013), and TNF-α level was found to be elevated in the midbrain of a PD mouse model; in fact, the initial oxidative insult in the induction of PD caused a loss of ventral midbrain DA neurons that triggered an inflammatory response (Srivastava et al., 2012). TNF-α promotes neurodegeneration by stimulating ROS production and release. In our study, TNF-α and Il-1β levels were increased in the brain of MPTP-treated mice relative to controls, which is in accordance with previous findings (Chung et al., 2010; Lofrumento et al., 2011). Interestingly, catalpol administration reversed the MPTP-induced decreases in SOD1, NLRX1, and GPX4 protein expression, indicating that the antioxidant defense capacity was enhanced. Moreover, catalpol reduced the levels of the proinflammatory cytokines TNF-α and IL-1β and NLRP3 inflammasome components. These results indicate that catalpol mitigates MPTP-induced oxidative stress to prevent chronic inflammation and neurodegeneration.

In summary, the results of this study demonstrate that catalpol treatment alleviates MPTP-induced degeneration of DA neurons *in vivo*. The neuroprotective effects of catalpol are at least partly due to the attenuation of inflammation, enhance antioxidant defense and reduce oxidative stress, and inhibition of apoptosis in DA neurons of the SN and striatum through downregulation of MKK4/JNK/c-Jun signaling. Our findings highlight the clinical potential of catalpol for the management of PD symptoms. The pre-treatment with candidate agents for PD intervention is limited by the specific biomarkers that could confirm the asymptomatic stage of PD. Extensive experiments, pre-clinical and clinical evaluation need to be performed.

## Data Availability Statement

All datasets generated for this study are included in the article/Supplementary Material.

## Ethics Statement

The animal study was reviewed and approved by The Laboratory Ethics Committee of China Medical University.

## Author Contributions

L-YW and XY conceived the study and designed the experiments. X-XL and Y-NZ performed the experiments. L-YW, XY and C-YW interpreted the data and wrote the manuscript. Z-YH critically reviewed the manuscript and obtained funding for the study. All authors have read and approved the final version of the manuscript.

## Conflict of Interest

The authors declare that the research was conducted in the absence of any commercial or financial relationships that could be construed as a potential conflict of interest.

## References

[B1] Allegra Mascaro A. L. Cesare P. Sacconi L. Grasselli G. Mandolesi G. Maco B.. (2013). *In vivo* single branch axotomy induces GAP-43-dependent sprouting and synaptic remodeling in cerebellar cortex. Proc. Natl. Acad. Sci. U S A 110, 10824–10829. 10.1073/pnas.1219256110 23754371 PMC3696745

[B2] Barcia C. Ros C. M. Annese V. Gómez A. Ros-Bernal F. Aguado-Yera D.. (2011). IFN-γ signaling, with the synergistic contribution of TNF-α, mediates cell specific microglial and astroglial activation in experimental models of Parkinson’s disease. Cell Death Dis. 2:e142. 10.1038/cddis.2011.17 21472005 PMC3122054

[B3] Bendor J. T. Logan T. P. Edwards R. H. (2013). The function of α-synuclein. Neuron 79, 1044–1066. 10.1016/j.neuron.2013.09.004 24050397 PMC3866954

[B4] Bi J. Jiang B. Hao S. Zhang A. Dong Y. Jiang T.. (2009). Catalpol attenuates nitric oxide increase via ERK signaling pathways induced by rotenone in mesencephalic neurons. Neurochem. Int. 54, 264–270. 10.1016/j.neuint.2008.12.003 19111870

[B5] Bi J. Jiang B. Liu J. H. Lei C. Zhang X. L. An L. J. (2008a). Protective effects of catalpol against H2O2-induced oxidative stress in astrocytes primary cultures. Neurosci. Lett. 442, 224–227. 10.1016/j.neulet.2008.07.029 18652878

[B6] Bi J. Wang X. B. Chen L. Hao S. An L. J. Jiang B.. (2008b). Catalpol protects mesencephalic neurons against MPTP induced neurotoxicity via attenuation of mitochondrial dysfunction and MAO-B activity. Toxicol in vitro 22, 1883–1889. 10.1016/j.tiv.2008.09.007 18840519

[B7] Cai Q. Ma T. Li C. Tian Y. Li H. (2016). Catalpol protects pre-myelinating oligodendrocytes against ischemia-induced oxidative injury through ERK1/2 signaling pathway. Int. J. Biol. Sci. 12, 1415–1426. 10.7150/ijbs.16823 27994507 PMC5166484

[B8] Carr A. C. McCall M. R. Frei B. (2000). Oxidation of LDL by myeloperoxidase and reactive nitrogen species: reaction pathways and antioxidant protection. Arterioscler. Thromb. Vasc. Biol. 20, 1716–1723. 10.1161/01.atv.20.7.1716 10894808

[B9] Chen X. Lan X. Roche I. Liu R. Geiger J. D. (2008). Caffeine protects against MPTP-induced blood-brain barrier dysfunction in mouse striatum. J. Neurochem. 107, 1147–1157. 10.1111/j.1471-4159.2008.05697.x 18808450 PMC3692355

[B10] Chung Y. C. Ko H. W. Bok E. Park E. S. Huh S. H. Nam J. H.. (2010). The role of neuroinflammation on the pathogenesis of Parkinson’s disease. BMB Rep. 43, 225–232. 10.5483/bmbrep.2010.43.4.225 20423606

[B11] da Silva J. A. Tecuapetla F. Paixão V. Costa R. M. (2018). Dopamine neuron activity before action initiation gates and invigorates future movements. Nature 554, 244–248. 10.1038/nature25457 29420469

[B12] Dauer W. Przedborski S. (2003). Parkinson’s disease: mechanisms and models. Neuron 39, 889–909. 10.1016/s0896-6273(03)00568-3 12971891

[B13] Derijard B. Hibi M. Wu I. H. Barrett T. Su B. Deng T.. (1994). JNK1: a protein kinase stimulated by UV light and Ha-Ras that binds and phosphorylates the c-Jun activation domain. Cell 76, 1025–1037. 10.1016/0092-8674(94)90380-8 8137421

[B14] Dinda B. Dinda M. Kulsi G. Chakraborty A. Dinda S. (2019). Therapeutic potentials of plant iridoids in Alzheimer’s and Parkinson’s diseases: a review. Eur. J. Med. Chem. 169, 185–199. 10.1016/j.ejmech.2019.03.009 30877973

[B15] Dobbs R. J. Charlett A. Purkiss A. G. Dobbs S. M. Weller C. Peterson D. W. (1999). Association of circulating TNF-α and IL-6 with ageing and parkinsonism. Acta Neurol. Scand. 100, 34–41. 10.1111/j.1600-0404.1999.tb00721.x 10416510

[B16] Du Y. Ma Z. Lin S. Dodel R. C. Gao F. Bales K. R.. (2001). Minocycline prevents nigrostriatal dopaminergic neurodegeneration in the MPTP model of Parkinson’s disease. Proc. Natl. Acad. Sci. U S A 98, 14669–14674. 10.1073/pnas.251341998 11724929 PMC64739

[B17] Duan W. Ladenheim B. Cutler R. G. Kruman I. I. Cadet J. L. Mattson M. P. (2002). Dietary folate deficiency and elevated homocysteine levels endanger dopaminergic neurons in models of Parkinson’s disease. J. Neurochem. 80, 101–110. 10.1046/j.0022-3042.2001.00676.x 11796748

[B18] Dunn C. Wiltshire C. MacLaren A. Gillespie D. A. (2002). Molecular mechanism and biological functions of c-Jun N-terminal kinase signalling via the c-Jun transcription factor. Cell. Signal. 14, 585–593. 10.1016/s0898-6568(01)00275-3 11955951

[B19] Duty S. Jenner P. (2011). Animal models of Parkinson’s disease: a source of novel treatments and clues to the cause of the disease. Br. J. Pharmacol. 164, 1357–1391. 10.1111/j.1476-5381.2011.01426.x 21486284 PMC3229766

[B20] Falschlehner C. Emmerich C. H. Gerlach B. Walczak H. (2007). TRAIL signalling: decisions between life and death. Int. J. Biochem. Cell Biol. 39, 1462–1475. 10.1016/j.biocel.2007.02.007 17403612

[B21] Fischer R. Maier O. (2015). Interrelation of oxidative stress and inflammation in neurodegenerative disease: role of TNF. Oxid. Med. Cell. Longev. 2015:610813. 10.1155/2015/610813 25834699 PMC4365363

[B22] Fiszer U. Mix E. Fredrikson S. Kostulas V. Olsson T. Link H. (1994). γδ+ T cells are increased in patients with Parkinson’s disease. J. Neurol. Sci. 121, 39–45. 10.1016/0022-510x(94)90154-6 8133310

[B23] Gao H. M. Hong J. S. Zhang W. Liu B. (2002). Distinct role for microglia in rotenone-induced degeneration of dopaminergic neurons. J. Neurosci. 22, 782–790. 10.1523/JNEUROSCI.22-03-00782.2002 11826108 PMC6758500

[B24] Ghavami S. Shojaei S. Yeganeh B. Ande S. R. Jangamreddy J. R. Mehrpour M.. (2014). Autophagy and apoptosis dysfunction in neurodegenerative disorders. Prog. Neurobiol. 112, 24–49. 10.1016/j.pneurobio.2013.10.004 24211851

[B25] Greenberg D. A. Jin K. (2005). From angiogenesis to neuropathology. Nature 438, 954–959. 10.1038/nature04481 16355213

[B26] Haddad J. J. (2002). Oxygen-sensitive pro-inflammatory cytokines, apoptosis signaling and redox-responsive transcription factors in development and pathophysiology. Cytokines Cell. Mol. Ther. 7, 1–14. 10.1080/13684730216401 12171246

[B27] Herrero M. T. Estrada C. Maatouk L. Vyas S. (2015). Inflammation in Parkinson’s disease: role of glucocorticoids. Front. Neuroanat. 9:32. 10.3389/fnana.2015.00032 25883554 PMC4382972

[B28] Hirsch E. C. Hunot S. (2009). Neuroinflammation in Parkinson’s disease: a target for neuroprotection? Lancet Neurol. 8, 382–397. 10.1016/s1474-4422(09)70062-6 19296921

[B29] Hu L. Sun Y. Hu J. (2010). Catalpol inhibits apoptosis in hydrogen peroxide-induced endothelium by activating the PI3K/Akt signaling pathway and modulating expression of Bcl-2 and Bax. Eur. J. Pharmacol. 628, 155–163. 10.1016/j.ejphar.2009.11.046 19962976

[B30] Imamura K. Hishikawa N. Sawada M. Nagatsu T. Yoshida M. Hashizume Y. (2003). Distribution of major histocompatibility complex class II-positive microglia and cytokine profile of Parkinson’s disease brains. Acta Neuropathol. 106, 518–526. 10.1007/s00401-003-0766-2 14513261

[B31] Jang H. Boltz D. A. Webster R. G. Smeyne R. J. (2009). Viral parkinsonism. Biochim. Biophys. Acta 1792, 714–721. 10.1016/j.bbadis.2008.08.001 18760350 PMC4642437

[B32] Jiang B. Zhang H. Bi J. Zhang X. L. (2008). Neuroprotective activities of catalpol on MPP+/MPTP-induced neurotoxicity. Neurol. Res. 30, 639–644. 10.1179/174313208x289543 18423111

[B33] Kalia L. V. Lang A. E. (2015). Parkinson’s disease. Lancet 386, 896–912. 10.1016/S0140-6736(14)61393-3 25904081

[B34] Kettenmann H. Kirchhoff F. Verkhratsky A. (2013). Microglia: new roles for the synaptic stripper. Neuron 77, 10–18. 10.1016/j.neuron.2012.12.023 23312512

[B35] Khan M. M. Kempuraj D. Thangavel R. Zaheer A. (2013). Protection of MPTP-induced neuroinflammation and neurodegeneration by Pycnogenol. Neurochem. Int. 62, 379–388. 10.1016/j.neuint.2013.01.029 23391521 PMC3604118

[B36] Kordower J. H. Olanow C. W. Dodiya H. B. Chu Y. Beach T. G. Adler C. H.. (2013). Disease duration and the integrity of the nigrostriatal system in Parkinson’s disease. Brain 136, 2419–2431. 10.1093/brain/awt192 23884810 PMC3722357

[B37] Lee D. H. Kim C. S. Lee Y. J. (2011). Astaxanthin protects against MPTP/MPP^+^-induced mitochondrial dysfunction and ROS production *in vivo* and *in vitro*. Food Chem. Toxicol. 49, 271–280. 10.1016/j.fct.2010.10.029 21056612 PMC3010303

[B38] Liu B. Gao H. M. Hong J. S. (2003). Parkinson’s disease and exposure to infectious agents and pesticides and the occurrence of brain injuries: role of neuroinflammation. Environ. Health Perspect. 111, 1065–1073. 10.1289/ehp.6361 12826478 PMC1241555

[B39] Liu C. L. Kershberg L. Wang J. Schneeberger S. Kaeser P. S. (2018). Dopamine secretion is mediated by sparse active zone-like release sites. Cell 172, 706.e15–718.e15. 10.1016/j.cell.2018.01.008 29398114 PMC5807134

[B40] Lofrumento D. D. Saponaro C. Cianciulli A. De Nuccio F. Mitolo V. Nicolardi G.. (2011). MPTP-induced neuroinflammation increases the expression of pro-inflammatory cytokines and their receptors in mouse brain. Neuroimmunomodulation 18, 79–88. 10.1159/000320027 20938211

[B41] Mao Y. R. Jiang L. Duan Y. L. An L. J. Jiang B. (2007). Efficacy of catalpol as protectant against oxidative stress and mitochondrial dysfunction on rotenone-induced toxicity in mice brain. Environ. Toxicol. Pharmacol. 23, 314–318. 10.1016/j.etap.2006.11.012 21783774

[B42] Mashima K. Takahashi S. Minami K. Izawa Y. Abe T. Tsukada N.. (2018). Neuroprotective role of astroglia in Parkinson disease by reducing oxidative stress through dopamine-induced activation of pentose-phosphate pathway. ASN Neuro 10:1759091418775562. 10.1177/1759091418775562 29768946 PMC5960859

[B43] Miyazaki I. Asanuma M. (2008). Dopaminergic neuron-specific oxidative stress caused by dopamine itself. Acta Med. Okayama 62, 141–150. 10.18926/AMO/30980 18596830

[B44] Moore D. J. West A. B. Dawson V. L. Dawson T. M. (2005). Molecular pathophysiology of Parkinson’s disease. Annu. Rev. Neurosci. 28, 57–87. 10.1146/annurev.neuro.28.061604.135718 16022590

[B45] Nomura D. K. Morrison B. E. Blankman J. L. Long J. Z. Kinsey S. G. Marcondes M. C.. (2011). Endocannabinoid hydrolysis generates brain prostaglandins that promote neuroinflammation. Science 334, 809–813. 10.1126/science.1209200 22021672 PMC3249428

[B46] Noyce A. J. Bestwick J. P. Silveira-Moriyama L. Hawkes C. H. Giovannoni G. Lees A. J.. (2012). Meta-analysis of early nonmotor features and risk factors for Parkinson disease. Ann. Neurol. 72, 893–901. 10.1002/ana.23687 23071076 PMC3556649

[B47] Ogata A. Tashiro K. Nukuzuma S. Nagashima K. Hall W. W. (1997). A rat model of Parkinson’s disease induced by Japanese encephalitis virus. J. Neurovirol. 3, 141–147. 10.3109/13550289709015803 9111176

[B48] Okabe K. Kobayashi S. Yamada T. Kurihara T. Tai-Nagara I. Miyamoto T.. (2014). Neurons limit angiogenesis by titrating VEGF in retina. Cell 159, 584–596. 10.1016/j.cell.2014.09.025 25417109

[B49] Perier C. Bové J. Wu D. C. Dehay B. Choi D. K. Jackson-Lewis V.. (2007). Two molecular pathways initiate mitochondria-dependent dopaminergic neurodegeneration in experimental Parkinson’s disease. Proc. Natl. Acad. Sci. U S A 104, 8161–8166. 10.1073/pnas.0609874104 17483459 PMC1876588

[B50] Petroske E. Meredith G. E. Callen S. Totterdell S. Lau Y. S. (2001). Mouse model of Parkinsonism: a comparison between subacute MPTP and chronic MPTP/probenecid treatment. Neuroscience 106, 589–601. 10.1016/s0306-4522(01)00295-0 11591459

[B51] Qin L. Wu X. Block M. L. Liu Y. Breese G. R. Hong J. S.. (2007). Systemic LPS causes chronic neuroinflammation and progressive neurodegeneration. Glia 55, 453–462. 10.1002/glia.20467 17203472 PMC2871685

[B52] Ransohoff R. M. (2016). How neuroinflammation contributes to neurodegeneration. Science 353, 777–783. 10.1126/science.aag2590 27540165

[B53] Ren Z. Yang N. Ji C. Zheng J. Wang T. Liu Y.. (2015). Neuroprotective effects of 5–(4-hydroxy-3-dimethoxybenzylidene)-thiazolidinone in MPTP induced Parkinsonism model in mice. Neuropharmacology 93, 209–218. 10.1016/j.neuropharm.2015.01.030 25680233

[B54] Santiago R. M. Barbieiro J. Lima M. M. Dombrowski P. A. Andreatini R. Vital M. A. (2010). Depressive-like behaviors alterations induced by intranigral MPTP, 6-OHDA, LPS and rotenone models of Parkinson’s disease are predominantly associated with serotonin and dopamine. Prog. Neuropsychopharmacol. Biol. Psychiatry 34, 1104–1114. 10.1016/j.pnpbp.2010.06.004 20547199

[B55] Scalzo P. Kümmer A. Cardoso F. Teixeira A. L. (2010). Serum levels of interleukin-6 are elevated in patients with Parkinson’s disease and correlate with physical performance. Neurosci. Lett. 468, 56–58. 10.1016/j.neulet.2009.10.062 19857551

[B56] Selvakumar G. P. Janakiraman U. Essa M. M. Justin Thenmozhi A. Manivasagam T. (2014). Escin attenuates behavioral impairments, oxidative stress and inflammation in a chronic MPTP/probenecid mouse model of Parkinson’s disease. Brain Res. 1585, 23–36. 10.1016/j.brainres.2014.03.010 24657313

[B57] Sriram K. Matheson J. M. Benkovic S. A. Miller D. B. Luster M. I. O’Callaghan J. P. (2006). Deficiency of TNF receptors suppresses microglial activation and alters the susceptibility of brain regions to MPTP-induced neurotoxicity: role of TNF-α. FASEB J. 20, 670–682. 10.1096/fj.05-5106com 16581975

[B58] Srivastava G. Dixit A. Yadav S. Patel D. K. Prakash O. Singh M. P. (2012). Resveratrol potentiates cytochrome P450 2 d22-mediated neuroprotection in maneb- and paraquat-induced parkinsonism in the mouse. Free Radic. Biol. Med. 52, 1294–1306. 10.1016/j.freeradbiomed.2012.02.005 22334051

[B59] Świątkiewicz M. Zaremba M. Joniec I. Członkowski A. Kurkowska-Jastrzębska I. (2013). Potential neuroprotective effect of ibuprofen, insights from the mice model of Parkinson’s disease. Pharmacol. Rep. 65, 1227–1236. 10.1016/s1734-1140(13)71480-4 24399718

[B60] Szabó C. Ischiropoulos H. Radi R. (2007). Peroxynitrite: biochemistry, pathophysiology and development of therapeutics. Nat. Rev. Drug Discov. 6, 662–680. 10.1038/nrd2222 17667957

[B61] Taylor T. N. Greene J. G. Miller G. W. (2010). Behavioral phenotyping of mouse models of Parkinson’s disease. Behav. Brain Res. 211, 1–10. 10.1016/j.bbr.2010.03.004 20211655 PMC2862121

[B62] Tian Y. Y. An L. J. Jiang L. Duan Y. L. Chen J. Jiang B. (2006). Catalpol protects dopaminergic neurons from LPS-induced neurotoxicity in mesencephalic neuron-glia cultures. Life Sci. 80, 193–199. 10.1016/j.lfs.2006.09.010 17049947

[B63] Tian Y. Y. Jiang B. An L. J. Bao Y. M. (2007). Neuroprotective effect of catalpol against MPP^+^-induced oxidative stress in mesencephalic neurons. Eur. J. Pharmacol. 568, 142–148. 10.1016/j.ejphar.2007.04.039 17512520

[B64] Verrecchia F. Tacheau C. Wagner E. F. Mauviel A. (2003). A central role for the JNK pathway in mediating the antagonistic activity of pro-inflammatory cytokines against transforming growth factor-β-driven SMAD3/4-specific gene expression. J. Biol. Chem. 278, 1585–1593. 10.1074/jbc.m206927200 12426318

[B65] Waetzig V. Czeloth K. Hidding U. Mielke K. Kanzow M. Brecht S.. (2005). c-Jun N-terminal kinases (JNKs) mediate pro-inflammatory actions of microglia. Glia 50, 235–246. 10.1002/glia.20173 15739188

[B66] Wahner A. D. Bronstein J. M. Bordelon Y. M. Ritz B. (2007). Nonsteroidal anti-inflammatory drugs may protect against Parkinson disease. Neurology 69, 1836–1842. 10.1212/01.wnl.0000279519.99344.ad 17984451

[B69] Wang S. He H. Chen L. Zhang W. Zhang X. Chen J. (2015). Protective effects of salidroside in the MPTP/MPP^+^-induced model of Parkinson’s disease through ROS-NO-related mitochondrion pathway. Mol. Neurobiol. 51, 718–728. 10.1007/s12035-014-8755-0 24913834

[B71] Wang Z. Huang X. Zhao P. Zhao L. Wang Z. Y. (2018). Catalpol inhibits amyloid-β generation through promoting α-cleavage of APP in swedish mutant APP overexpressed N2a Cells. Front. Aging Neurosci. 10:66. 10.3389/fnagi.2018.00066 29615891 PMC5867310

[B68] Wang Q. Xing M. Chen W. Zhang J. Qi H. Xu X. (2012). HPLC-APCI-MS/MS method for the determination of catalpol in rat plasma and cerebrospinal fluid: application to an *in vivo* pharmacokinetic study. J. Pharm. Biomed. Anal. 70, 337–343. 10.1016/j.jpba.2012.05.016 22677654

[B67] Wang N. Yang W. Xiao T. Miao Z. Luo W. You Z.. (2018). Possible role of miR-204 in optic nerve injury through the regulation of GAP-43. Mol. Med. Rep. 17, 3891–3897. 10.3892/mmr.2017.8341 29286154

[B70] Wang Z. H. Zhan-Sheng H. (2018). Catalpol inhibits migration and induces apoptosis in gastric cancer cells and in athymic nude mice. Biomed. Pharmacother. 103, 1708–1719. 10.1016/j.biopha.2018.03.094 29864961

[B72] Whitfield J. Neame S. J. Paquet L. Bernard O. Ham J. (2001). Dominant-negative c-Jun promotes neuronal survival by reducing BIM expression and inhibiting mitochondrial cytochrome c release. Neuron 29, 629–643. 10.1016/s0896-6273(01)00239-2 11301023

[B73] Wu D. C. Jackson-Lewis V. Vila M. Tieu K. Teismann P. Vadseth C.. (2002). Blockade of microglial activation is neuroprotective in the 1-methyl-4-phenyl-1,2,3,6-tetrahydropyridine mouse model of Parkinson disease. J. Neurosci. 22, 1763–1771. 10.1523/JNEUROSCI.22-05-01763.2002 11880505 PMC6758858

[B74] Xu G. Xiong Z. Yong Y. Wang Z. Ke Z. Xia Z.. (2010). Catalpol attenuates MPTP induced neuronal degeneration of nigral-striatal dopaminergic pathway in mice through elevating glial cell derived neurotrophic factor in striatum. Neuroscience 167, 174–184. 10.1016/j.neuroscience.2010.01.048 20123001

[B75] Yan J. Wang C. Jin Y. Meng Q. Liu Q. Liu Z.. (2018). Catalpol ameliorates hepatic insulin resistance in type 2 diabetes through acting on AMPK/NOX4/PI3K/AKT pathway. Pharmacol. Res. 130, 466–480. 10.1016/j.phrs.2017.12.026 29284152

[B76] Zhang X. Jin C. Li Y. Guan S. Han F. Zhang S. (2013). Catalpol improves cholinergic function and reduces inflammatory cytokines in the senescent mice induced by D-galactose. Food Chem. Toxicol. 58, 50–55. 10.1016/j.fct.2013.04.006 23612000

[B78] Zhang Y. Wang C. Jin Y. Yang Q. Meng Q. Liu Q.. (2018). Activating the PGC-1α/TERT pathway by catalpol ameliorates atherosclerosis via modulating ROS production, DNA damage, and telomere function: implications on mitochondria and telomere link. Oxid. Med. Cell. Longev. 2018:2876350. 10.1155/2018/2876350 30046372 PMC6036816

[B77] Zhang Y. H. Wang D. W. Xu S. F. Zhang S. Fan Y. G. Yang Y. Y.. (2018). α-Lipoic acid improves abnormal behavior by mitigation of oxidative stress, inflammation, ferroptosis, and tauopathy in P301S Tau transgenic mice. Redox Biol. 14, 535–548. 10.1016/j.redox.2017.11.001 29126071 PMC5684493

[B79] Zhou J. Xu G. Ma S. Li F. Yuan M. Xu H.. (2015). Catalpol ameliorates high-fat diet-induced insulin resistance and adipose tissue inflammation by suppressing the JNK and NF-κB pathways. Biochem. Biophys. Res. Commun. 467, 853–858. 10.1016/j.bbrc.2015.10.054 26474703

[B80] Zou H. Henzel W. J. Liu X. Lutschg A. Wang X. (1997). Apaf-1, a human protein homologous to C. elegans CED-4, participates in cytochrome c-dependent activation of caspase-3. Cell 90, 405–413. 10.1016/s0092-8674(00)80501-2 9267021

