# Supplementary material for: Catalpol Exerts a Neuroprotective Effect in the MPTP Mouse Model of Parkinson’s Disease
Source: Front Aging Neurosci. 2019 Nov 15;11:316. doi: 10.3389/fnagi.2019.00316 (PMC6889905; doi:10.3389/fnagi.2019.00316)
Supplement: Supplementary file 1 [file Data_Sheet_1.DOCX]

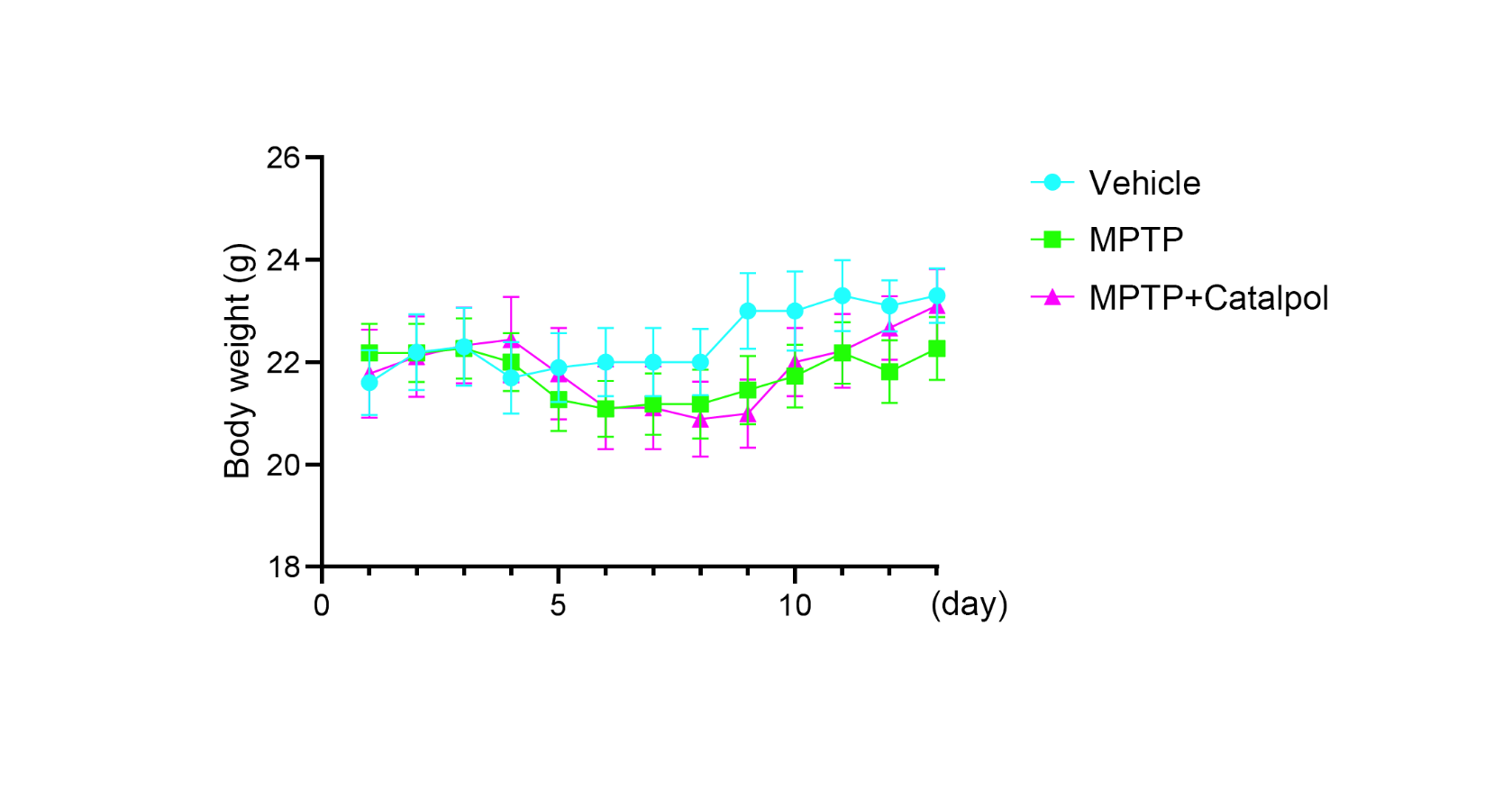


**Supplement 1. Daily body weight monitoring of the mice under vehicle, MPTP or MPTP+catalpol treatment.** MPTP or catalpol treatment did not markedly affect the body weight of the mice. Data represent mean ± SEM; n = 9-11 mice per group.
